# Lonicera japonica Thunb extract ameliorates lipopolysaccharide-induced acute lung injury associated with luteolin-mediated suppression of NF-κB signaling pathway

**DOI:** 10.1186/s12950-023-00372-9

**Published:** 2023-12-19

**Authors:** Qinyao Jia, Jing wen, Qi Yang, Shengming Liu, Jianwu Zhang, Tao Wang, Yao Cheng

**Affiliations:** 1 School of Pharmacy, North Sichuan Medical College, Nanchong, People’s Republic of China; 2 Department of Tuberculosis, Chengdu Public Health Clinical Medical Center, Chengdu, People’s Republic of China; 3 Department of Pulmonary and Critical Care Medicine, University of Chinese Academy of Sciences Shenzhen Hospital, Shenzhen & The first Affiliated Hospital of Jinan University, Guangzhou, People’s Republic of China; 4 Department of Pulmonary and Critical Care Medicine, Affiliated Hospital of North Sichuan Medical College, Nanchong, People’s Republic of China; 5 Department of Pulmonary and Critical Care Medicine, The First Affiliated Hospital of Jinan University, Guangzhou, People’s Republic of China

**Keywords:** LJT, LTE, Lut, ALI, NF-κB

## Abstract

**Objective:**

Lonicera japonica Thunb (LJT) is a commonly used herbal soup to treat inflammation-related diseases. However, the effect of LJT on ALI is unknown. The present study was aimed at investigating the protective effects of LJT extract (LTE) and its active ingredient luteolin (Lut) on lipopolysaccharide (LPS)-stimulated ALI and investigate its potential mechanism.

**Materials and methods:**

The effects of LTE and Lut were explored in an ALI mouse model induced by intraperitoneal injection of lipopolysaccharide (LPS). Besides, the LPS-induced inflammation model in BEAS-2B cells was used to clarify the underlying mechanisms. The ALI pathological changes in lung tissues were tested through Haematoxylin and eosin (HE) staining. The apoptosis of cells in lung tissue and the cell model in vitro was evaluated by TUNEL assays, respectively. Meanwhile, the viability of cells in vitro was evaluated by Cell Counting Kit-8 (CCK-8) assay. The levels/concentrations of tumor necrosis factor-α (TNF-α), interleukin-6 (IL-6), IL-1β and IL-10 in BALF were detected by enzyme-linked immunosorbent assay (ELISA). Besides, through quantitative real-time polymerase chain reaction (qRT-PCR) and Western blotting, the expression of the above-mentioned inflammatory factors and key factors in the NF-κB signaling pathway was examined. The distribution of inflammatory factors in tissue was observed through immunohistochemistry (IHC) assays .

**Results:**

In relative to LPS-stimulated group, the in vivo study showed that LTE and different concentrations of Lut dramatically alleviated LPS-evoked lung pathological injury and lung edema based on the changes in total protein levels and lung wet/dry (W/D) ratio in the bronchoalveolar lavage fluid (BALF) from ALI mice. LTE and different concentrations of Lut also suppressed the inflammatory response, as reflected by the variations of neutrophil accumulation and the production of proinflammatory and anti-inflammatory cytokines in the lung tissues and BALF of ALI mice. The in vitro research also demonstrated that LTE and Lut visibly facilitated cell viability and restrained the apoptosis of BEAS-2B cells stimulated by LPS. Lut hindered LPS-inducible activation of NF-κB pathway in BEAS-2B cells.

**Conclusion:**

The present study proved that LTE might suppress LPS-induced acute injury and inflammation in mice and BEAS-2B cells through the Lut-caused suppression of NF-κB signal path (Figure 1).

**Supplementary Information:**

The online version contains supplementary material available at 10.1186/s12950-023-00372-9.

## Introduction

Acute lung injury (ALI) refers to a serious respiratory disorder featured by acute hypoxic respiratory failure, diffuse pulmonary infiltration, and pulmonary edema resulting from non-left atrial hypertension, or non-cardiogenic pulmonary edema [1]. The end-stage ALI exhibits high mortality and is one kind respiratory syndrome caused by pneumonia, sepsis, trauma, burns, and inhalation of harmful gases [2, 3]. The occurrence of ALI results in the injury of alveoli, inflammatory cell infiltration and uncontrollable inflammation [4, 5]. The epidermic cells of alveoli exert vital function in ALI through the apoptosis and continuous development of inflammation [6, 7]. Despite continuous advances in clinical treatment strategies for ALI, the death rate of ALI patients is still high and the efficient therapeutic drug is lacking in the interference of ALI [8]. Therefore, it is urgently needed to further explore the etiopathogenesis of ALI and discover novel effective curative agents for the treatment of ALI.

**Fig. 1:**
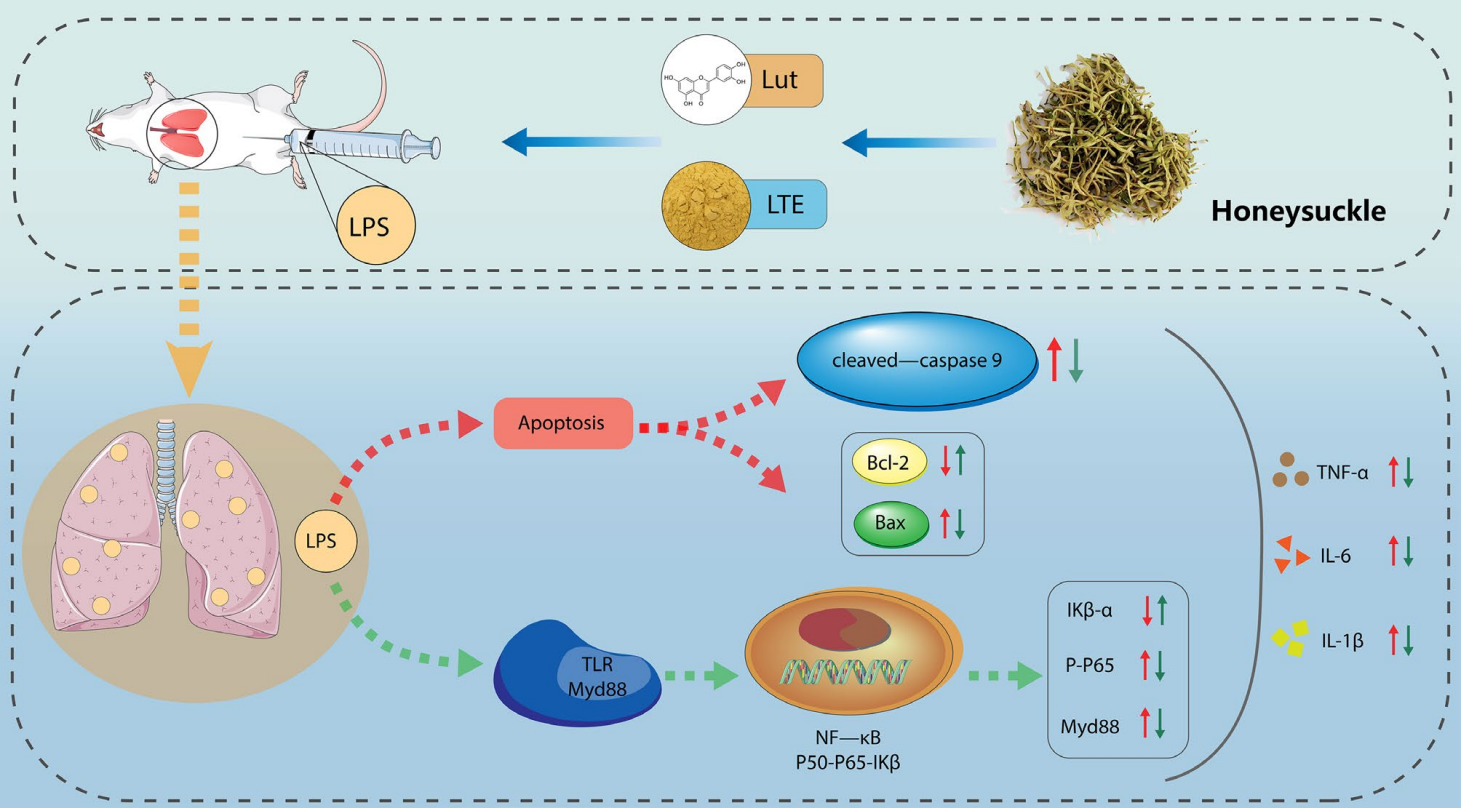
The protective effect of LTE/Lut on LPS-induced acute lung injury and the underlying mechanism. Note: LTE might suppress LPS-induced acute injury and inflammation in mice and BEAS-2B cells through the Lut-caused suppression of NF-κB signal path

Numerous studies have suggested that gram-negative bacterial infections are among the most important causes of ALI, and lipopolysaccharide (LPS) can result in lung injury and inflammatory response, which is the major component of the outer membrane of gram-negative bacteria [9–11]. In recent years, LPS-induced ALI has been the most widely used research method, which can effectively trigger a neutrophilic inflammatory response with increased levels of intrapulmonary cytokines. Directly or indirectly damages pulmonary vascular endothelial cells and alveolar epithelial cells, which causes enhanced pulmonary capillary permeability, inflammatory cell infiltration and large-scale release of inflammation mediators, ultimately resulting in lung injury.

Louicera japonica Thunb (LJT), which is an “antidote” in Chinese traditional herbal medicines, is adopted for the treatment of “lung heat cough”. In clinical practice, various Chinese patent medicine injections including LJT and the extracts are widely utilized for treating respiration-related disorders, which have made favorable curative effect. Louicera japonica Thunb extract (LTE) is rich in various chemical components, mainly for organic acids, including, Lut, chlorogenic acid, isochlorogenic acid, caffeic acid and palmitic acid [12]. Among them, Lut is a typical kind of natural flavonoid compound and an effective active monomer with strong oxidative capacity [13]. Modern studies have indicated that LTE has biological functions including antipyretic, anti-inflammatory, antibacterial, antiviral, immunomodulation and antioxidant. Similarly, as the major active ingredient of LTE, Lut was also verified to attenuate LPS-evoked injury in mice [14]. However, whether LTE can inhibit lung inflammation or other biological pathways to alleviate ALI through its active component Lut has not been fully covered. Furthermore, studies have shown that Decursin could mitigate LPS-evoked BEAS-2B and HPAEC cell injury via inactivating NF-κB pathway [15], and Chikusetsusaponin V could protect against LPS-stimulated ALI in mice by restraining NF-κB pathway activation [16]. However, whether the function of LTE in LPS-evoked ALI was concerned with NF-κB pathway remained unclear. Here, we explored the impact of LTE on LPS-provoked ALI cell and mouse models, and further confirmed whether the function of Lut was similar to LTE.

In the present study, we mainly analyzed that LTE protected LPS-provoked ALI mice and cell models, and investigated the possible mechanisms of LTE by gateway analyse of Gene Ontology (GO) and Kyoto Encyclopedia of Genes and Genomes (KEGG), confirming that LTE might inhibit LPS-induced injury through active ingredient Lut by regulating NF-κB signaling. Moreover, this study may provide novel therapeutic directions for ALI.

## Materials and methods

### Cell culture

Human alveolar epithelial cells (BEAS-2B) were provided by procell (Wuhan, China) and cultivated in DMEM (Solarbio, Beijing, China) that contained 1% antibiotics and 10% fetal bovine serum (FBS) (Solarbio) under 37 °C with 5% CO_2_. To construct ALI cell model, BEAS-2B cells (1.0 × 10^4^/well) were inoculated in the 96-well plates, followed by 12 h treatment with 10 µg/mL LPS or PBS (Solarbio) [17]. For drug treatment group, cells were treated with LTE at 10 ug/mL (Xian Musen Bioengineering, Xian, China) or Lut (Solarbio) at 10 µM for 24 h and then stimulated with LPS(10 µg/mL, 12 h) [17, 18].

### Establishment of the ALI mouse model

The approval of animal experimental protocols was provided by Animal Care and Use Committee of Shenzhen Hospital of University of Chinese Academy of Sciences(LL-KT-2,021,059). We obtained 6-8-week-old male C57BL/6 mice (20–30 g) in SPF (Beijing) Biotechnology Co., Ltd. (Beijing, China). Mice model was classified into two parts. In the first part, 24 mice were randomly classified into three groups (n = 8/group): normal (Sham), ALI and ALI + LTE groups. The survival experiments were also carried out in another three group mice (n = 8/group): normal (Sham), ALI and ALI + LTE groups. Survival of mice was monitored for 48 h. In the second part, 40 mice were further assigned into five groups (n = 8/group) randomly: Sham, ALI, ALI + Lut_L_ (18 µmol/kg Lut), ALI + Lut_M_ (35 µmol/kg Lut) and ALI + Lut_H_ (70 µmol/kg Lut) groups. The survival experiments were also performed in another five group mice (n = 8/group): Sham, ALI, ALI + Lut_L,_ ALI + Lut_M_ and ALI + Lut_H_ groups. Survival of mice was monitored for 48 h. With free access to food and water, mice were routinely housed in a specific animal room (24 ± 1 °C; 12 h/12 h light/dark cycle).

**Fig. 2:**
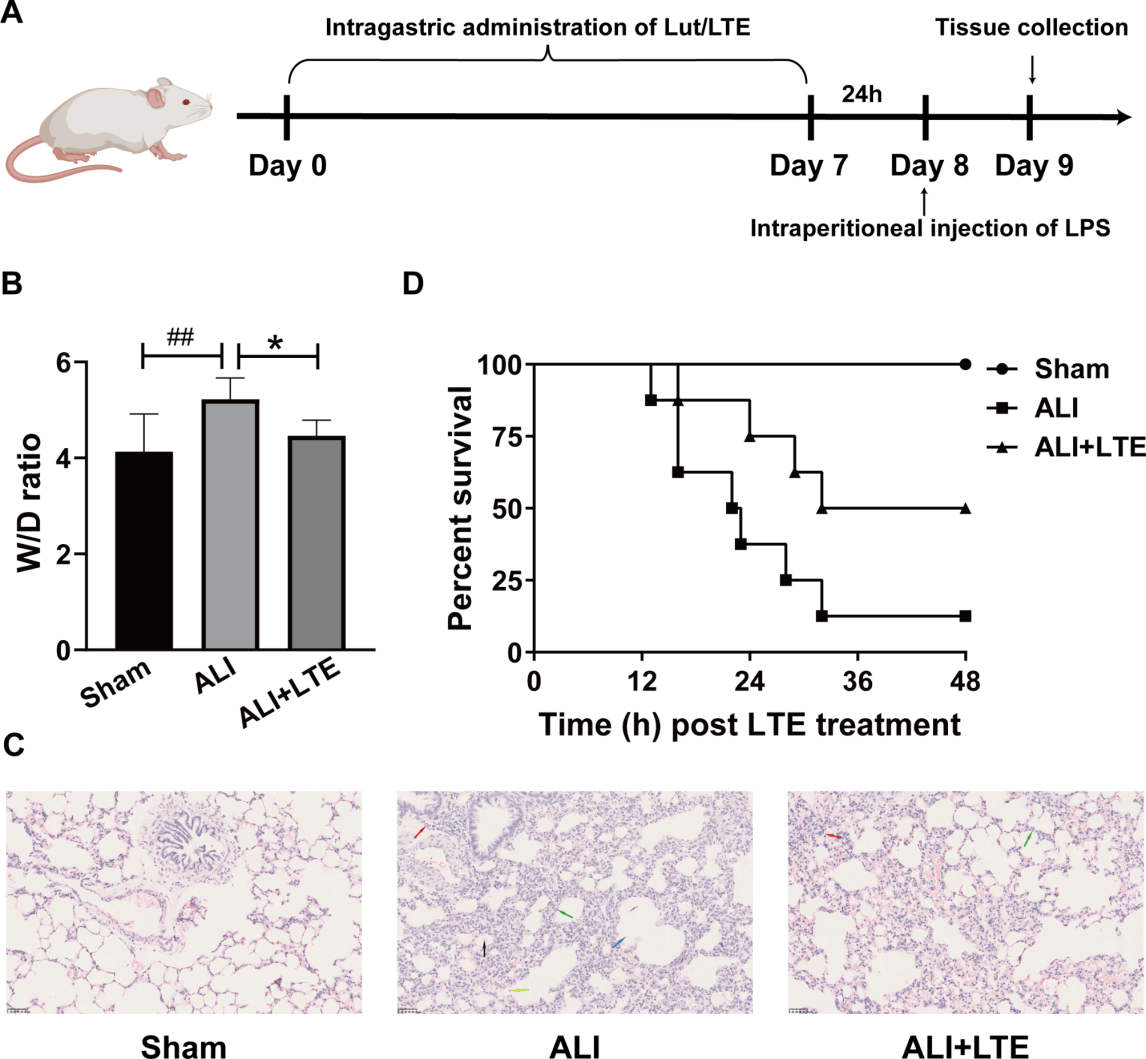
The effects of LTE on the survival rates and lung structure in LPS-induced ALI in mice. Note: (**A**) Schematic representation of the construction of ALI animal model and LTE/Lut administration. (**B**) Lung W/D weight ratio was determined in experimental groups. (**C**) The changes of histological structure of the mouse lung in different groups (20×). HE stainings indicate inflammatory cell infiltration (red arrows), edema (blue arrows), hemorrhage (black arrows), alveolar septal thickening (green arrows) and alveolar epithelial cell shedding (yellow arrows) in sections of the lung. (**D**) Survival curves in Sham group, ALI and ALI + LTE groups treated with control saline or LTE in mice. Natural death time was recorded by observation every half an hour starting 12 h after LPS. With the use of the Prism software, the death time was shown as a Kaplan-Meier plot. Log-rank test was adopted for comparing survival between groups. Other mice were harvested at 12 h post LPS and lung tissues harvested for analyses of histomorphology and W/D weight ratio were employed to evaluate severity of ALI. Bar = 50 μm. ^##^*p* < 0.01 vs. Sham group; *p < 0.05 vs. ALI model group

In order to establish ALI model, LPS (5 mg/kg) dissolved in sterile saline was intraperitoneally injected. Normal mice were given isodose saline through intragastrically injected for 7 consecutive days [10]. The entire experimental process is depicted in Fig. 2A. The mice in ALI + LTE, ALI + Lut_L,_ ALI + Lut_M_ and ALI + Lut_H_ group were intragastrically injected with a single dose of LTE (1.75 g/kg,) or Lut (18 µmol/kg, 35 µmol/kg and 70 µmol/kg) for 7 days before LPS treatment (5 mg/kg) [19, 20]. Each mouse was euthanized to remove lung tissue after 12 h and placed in -80 °C for subsequent experiments. In order to analyze the levels of protein and inflammatory cytokines, the left and right lungs of the mice were used to obtain lung wet-to-dry (W/D) weight ratio and bronchoalveolar lavage Fluid (BALF).

### The analysis of survival rates

To determine the influence of LTE or Lut on the survival time of mice with LPS infection, the mice were classified into different groups. The survival time of the mice was recorded daily. The day after the end of monitoring, all live mice in different groups were euthanized. To explore the survivorship curve, the time of natural death of mice was recorded after observing the survival status every 12 h after LPS treatment. Prism software was adopted for analyzing the survival curve, which was shown as Kaplan-Meier plot.

### Lung W/D weight ratio

To reflect severity degree of elevated endothelial permeability and damage of lungs, we determined lung W/D ratio, which could be an index of pneumonedema. After LPS treatment for 12 h, right lung tissues were gathered and weighed promptly after the removal (wet weight), and later put into oven for 48 h at 80 °C. To calculate dry weight, the lung after dehydration was reweighed once more. The lung weight rates before and after desiccation (wet/dry) were computed.

### Protein quantification and cytokines analysis in BALF

The lungs of mice were lavaged of sterile precooled PBS for three times through a tracheal cannula to harvest BALF solution. Then, BALF solution was subjected to 10 min centrifugation under 4 °C using centrifuge at 3000 rpm in order to obtain supernatant. The supernatants without cells were gathered and split into two portions to measure total protein levels using BCA protein assay kit (Beyotime) and inflammatory cytokines, respectively.

### Enzyme-linked immunosorbent assay (ELISA)

After 12 h of LPS or PBS treatment, BEAS-2B cells were planted into 96-well plates. Later, TNF-α, IL-1β, IL-6, and IL-10 contents in BEAS-2B cell supernatants and BALF solution were tested with relevant ELISA kits (Solarbio) in line with the specification. Following the standard instructions, the 96-well plates were recorded using a microplate reader. Based on standard curve, the absolute contents of the above factors were identified.

### Haematoxylin and eosin (HE) staining

The right lung tissues were gathered rapidly and fastened in 10% formalin overnight after LPS treatment for 12 h. Then, tissues were subjected to the dehydration and later embedded in paraffin. Subsequently, the sections were dyed with HE staining solution (Solarbio) to observe pathological changes of the alveolae and alveolar interstitial structure in mouse lung tissues using the light microscope.

### Immunohistochemistry (IHC) assay

The paraffin-embedded sections of lung tissues from mice were cut into slices (4 μm). Following antigen retrieval and washing, the sections were reacted with specified serum proteins and stained with primary antibodies against interleukin-6 (IL-6, ab208113, 1:50, Abcam, Cambridge, UK), IL-1β (ab283818, 1:500, Abcam), and tumor necrosis factor-α (TNF-α, ab6671, 1:1000, Abcam) for one night under 4℃. Subsequently, HRP-conjugated goat anti-rabbit secondary antibody (ab205718, 1:20000, Abcam) was supplemented to incubate slices for 1 h under room temperature. Next, color reaction was developed using diaminobenzidine. Nnuclei were counterstained with hematoxylin (Beyotime). Finally, typical areas were photographed under the light microscope at a suitable magnification.

### Quantitative real-time PCR (qRT-PCR)

Using TRIzol, total lung tissue and cellular RNAs were separated (Invitrogen, Carlsbad, CA, USA). Afterwards, cDNA template was prepared from extracted RNA using PrimeScript RT Reagent Kit (Takara, Dalian, China) via reverse transcription. Later, BeyoFast™ SYBR Green qPCR Mix (Beyotime) was utilized for qRT-PCR on a PCR system. GAPDH served as inner contrast. The sequences of specific Homo sapiens (human) and Mus musculus (mouse) primers were designed and listed in Supplementary Table 1. The relative expression of above inflammatory or anti-inflammatory factors was analyzed using 2^−ΔΔCt^ strategy, with β-actin being an inner reference. The primers were displayed in Table 1.

**Table 1:** The list of primer sequences for PCR

Name		Primers for PCR (5’-3’)
mouse-IL-1β	Forward	TGGACCTTCCAGGATGAGGACA
	Reverse	GTTCATCTCGGAGCCTGTAGTG
mouse-IL-6	Forward	TACCACTTCACAAGTCGGAGGC
	Reverse	CTGCAAGTGCATCATCGTTGTTC
mouse-IL-10	Forward	CGGGAAGACAATAACTGCACCC
	Reverse	CGGTTAGCAGTATGTTGTCCAGC
mouse-TNF-α	Forward	CTTCTCATTCCTGCTTGTG
	Reverse	ACTTGGTGGTTTGCTACG
mouse-Bax	Forward	AGGATGCGTCCACCAAGAAGCT
	Reverse	TCCGTGTCCACGTCAGCAATCA
mouse-Bcl-2	Forward	CCTGTGGATGACTGAGTACCTG
	Reverse	AGCCAGGAGAAATCAAACAGAGG
mouse-GAPDH	Forward	GGTTGTCTCCTGCGACTTCA
	Reverse	TGGTCCAGGGTTTCTTACTCC
human-Bax	Forward	TCAGGATGCGTCCACCAAGAAG
	Reverse	TGTGTCCACGGCGGCAATCATC
human-Bcl-2	Forward	ATCGCCCTGTGGATGACTGAGT
	Reverse	GCCAGGAGAAATCAAACAGAGGC
human-Cleaved-caspase9	Forward	CGCCATATCTAGTTTGCCCA
	Reverse	TCCGGAGGAAATTAAAGCAAC
human-MyD88	Forward	GAGGCTGAGAAGCCTTTACAGG
	Reverse	GCAGATGAAGGCATCGAAACGC
human-IκB-α (NFKBIA)	Forward	TCCACTCCATCCTGAAGGCTAC
	Reverse	CAAGGACACCAAAAGCTCCACG
human-NF-kB p65	Forward	TGAACCGAAACTCTGGCAGCTG
	Reverse	CATCAGCTTGCGAAAAGGAGCC
human-GAPDH	Forward	GTCTCCTCTGACTTCAACAGCG
	Reverse	ACCACCCTGTTGCTGTAGCCAA

### Western blot

The total protein was extracted utilizing RIPA buffer (Solarbio), separated via SDS-PAGE gel (10%) and transferred to PVDF membrane (Beyotime). Subsequently, 5% slim milk was used to block membranes under indoor environment for 1 h, followed by reaction with primary antibodies against B-cell lymphoma-2 (Bcl2, 26 kDa, ab182858, 1:1000, Abcam), Bcl-2-associated X protein (Bax, 21 kDa, ab32503, 1:1000, Abcam), IL-6 (23 kDa, ab208113, 1:1000, Abcam), IL-1β (31 kDa, ab283818, 1:1000, Abcam), TNF-α (26 kDa, ab6671, 1:1000, Abcam), IL-10 (20 kDa, ab33471, 1:1000, Abcam), medullary differentiation protein 88 (Myd88, 33 kDa, ab133739, 1:5000, Abcam), Phospho-nuclear factor kappaB (NF-kB) p65 (p-p65, 65 kDa, 3033T, 1:1000, Cell Signaling Technology, Danvers, Massachusetts, USA), NF-kB inhibitor alpha (IκB-α, 36 kDa, AI096, 1:1000, Beyotime), Cleaved-caspase9 (35 kDa, 9505T, 1:1000, Cell Signaling Technology), and internal reference GAPDH (36 kDa, ab181602, 1:10000, Abcam) overnight at 4℃. Subsequently, Rabbit IgG H&L (HRP) secondary antibody (ab205718, 1:10000, Abcam) or Rat IgG(H + L) (HRP) secondary antibody (SA00001-15, 1:2000, proteintech, Wuhan, China) was applied to interact with the membrane for 2 h in indoor environment. Thereafter, BeyoECL Star Kit (Beyotime) was used to visualize the immunoblots.

### TUNEL staining assay

The apoptotic capacity of BEAS-2B cells was analyzed using TUNEL staining (Roche, Basel, Switzerland). Afterwards, the sample slices were incubated for 30 min using proteinase K under 37 °C following deparaffinization and rehydration. Slices were rinsed by PBS twice before being placed in the TUNEL solution mixture at room temperature for 1 h. Later, the sections were dyed using 50 µL DAB for 10 min and redyed with hematoxylin for a couple of seconds. The apoptotic cells were stained green. The results about the representative stained pictures were presented under fluorescence microscope.

### Cell counting kit-8 (CCK-8) assay

To assess BEAS-2B cell viability, CCK-8 assay was performed. Briefly, BEAS-2B cells (5 × 10^4^/mL) were planted into the 96-well plate under 37 °C, followed by LPS treatment or not for 12 h. After being cultivated for specific time, 5 mg/mL CCK-8 solution (10 µL; Beyotime) was introduced into every well at 37℃ for 4 h-reaction. Finally, a microplate reader was adopted for measuring absorption (OD) values at 450 nm.

### Establishment of the compound-target-disease network and pathway enrichment analysis

Traditional Chinese Medicine Systems Pharmacology Database and Analysis Platform (TCMSP) and UniProt database (https://www.uniprot.org) were utilized for screening the volatile components of LJT and predicting the target genes of the volatile components in LTE, respectively. Genecards (https://www.genecards.org/) and OMIM (https://omim.org/) database were employed to obtain the target genes associated with ALI. The interaction network of ALI-LTE-component-target was established by Cytoscape 3.7.2 software. In addition, GO and KEGG analysis was carried out on mRNAs with differential expression using KEGG and R software package.

### Statistical analysis

All data were indicated by mean ± standard deviation (SD) and explored with the application of GraphPad Prism software. All assays were performed at least 3 replicates. The differences were compared via Student’s t-test or One-way ANOVA. *P* < 0.05 was thought to be of statistical significance.

## Results

### LTE mitigated LPS-induced ALI in vivo and improved survival of mice

Lung W/D ratio can indicate lung tissue edema, and it was evaluated in LPS-evoked mice. Prospectively, LPS-stimulated ALI model mice had significantly elevated lung W/D ratio in relative to the Sham group, which was substantially reduced by LTE among LPS-treated ALI mice (Fig. 2B). To evaluate the protection of LTE against LPS-induced ALI, morphological alterations in mouse lungs of LPS-stimulated mice were identified by HE staining. As displayed in Fig. 2C, the lung tissue alveolar wall structure of control (Sham) mice was intact, while after 12 h of LPS treatment, vascular leakiness substantially increased, as evidenced by interstitial/alveolar hemorrhage, edema, inflammatory cell infiltration and alveolar septal thickening in ALI mouse lung tissues compared with Sham group. All these features verified that the model of ALI was constructed. However, all above lung injuries induced by LPS were significantly inhibited by LTE pretreatment (Fig. 2C). To evaluate the latent differential role of LTE in regulating survival outcomes of LPS-evoked ALI mice, the survival curves were explored by calculating the living mouse numbers in diverse groups at 48 h following LPS. As displayed in Fig. 2D, the LPS-induced ALI mice exhibited poorer outcomes, with survival rates at 37.5% compared with sham-operated mice (100%) at 24 h. However, LTE treatment distinctly increased survival rate in LPS-stimulated ALI mice (75%). The obtained findings suggested that LTE could upregulate pulmonary hyperpermeability during LPS-mediated ALI.

### LTE’s anti-inflammatory effect in LPS-induced ALI mice

**Fig. 3:**
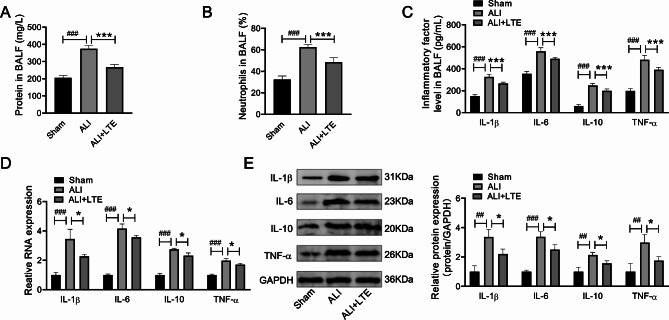
The protective impacts of LTE on the total protein concentration, the ratio of neutrophils in BALF and the expression of inflammatory factors in BALF and tissues of LPS-induced ALI mice. Note: 12 h after LPS challenge, (**A**) the total protein concentration, (**B**) the ratio of neutrophils and (**C**) the contents of IL-1β, IL-6, IL-10 and TNF-α in the supernatant of BALF were collected for analysis and were determined by kits. (**D**) The mRNA expression levels of IL-1β, IL-6, IL-10 and TNF-α in the tissues of mice were evaluated by qRT-PCR. (E)The protein expression levels of IL-1β, IL-6, IL-10 and TNF-α in the tissues of mice were evaluated by western blot assays. ^##^*p* < 0.01 and ^###^*p* < 0.001 vs. Sham group; *p < 0.05 and ***p < 0.001 vs. ALI model group

In comparison with Sham mice, ALI model mice might cause significant alterations of total protein content and neutrophil ratio in BALF, while LTE significantly reduced the effects, respectively (Fig. 3A and B). LTE’s role in inflammatory factor levels of ALI group were determined. As depicted in Fig. 3C, in relative to Sham group, LPS treatment in mice model resulted in significant changes of inflammatory factors in BALF, including enhancive IL-1β, IL-6, IL-10 and TNF-α contents. However, LTE significantly reduced the above factor levels in BALF versus ALI model mice (Fig. 3C). The impacts of LTE on inflammatory factors at protein and mRNA levels in ALI tissues were also evaluated. The results suggested that the expression of the above factors of mRNA and protein expression was notably enhanced in the tissues of ALI relative to the Sham group, whereas these effects were mitigated by LTE addition (Fig. 3D and E, Supplementary Table 1). These data suggested that LTE could prevent the progress of ALI triggered by LPS in mice.

### Screening for bioactive compounds and related targets of LTE in the treatment of ALI

**Fig. 4:**
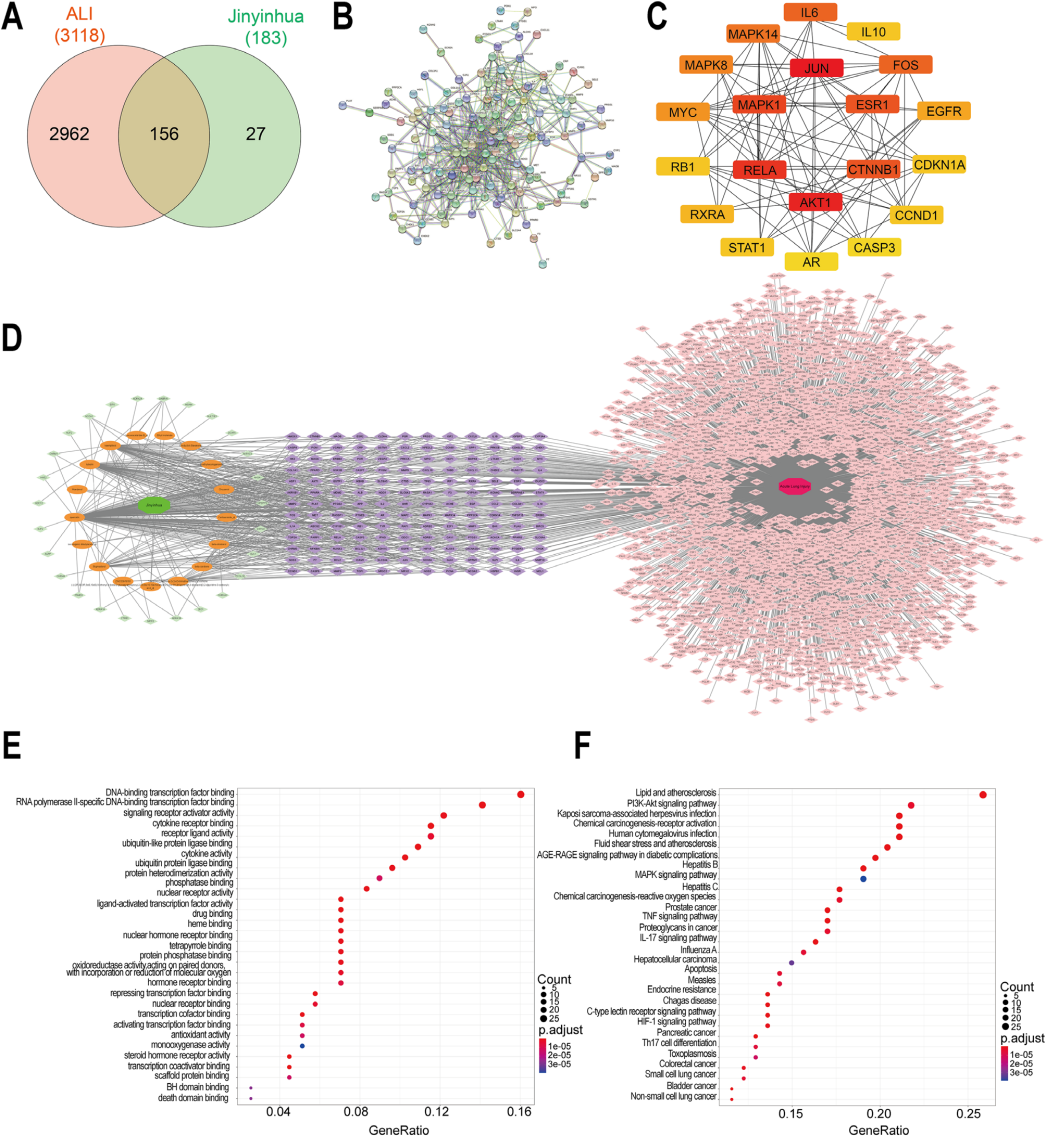
Screening for bioactive compounds and related targets of LTE in treating ALI. Note: (**A**) Venn diagram of the targets related to ALI and volatile components of LJT. (**B**) Protein-protein interaction network of potential target genes. (**C**) Top 20 potential core target genes based on Degree. (**D**) ALI-LJT-active ingredient-target interaction network. (**E**) GO annotation of the targets associated with ALI and volatile components of LJT. (**F**) KEGG pathway analysis of the signaling pathways correlated with ALI

After searching and screening the TCMSP database, 17 active ingredients of LJT were obtained. Totally 183 potential drug targets were obtained after prediction, combination and the removal of duplication. By searching GeneCards and OMIM databases, 3115 and 27 targets associated with ALI were obtained, respectively. After merging and removing duplication, 3118 targets associated with ALI were obtained. The obtained 183 drug active ingredient targets and 3118 disease targets related to ALI were imported into InteractiVenn website to obtain the Venn diagram. Meanwhile, 156 potential targets of LTE in the treatment of ALI were acquired by matching and mapping. Venn diagram representing the effective components of drugs and common drug-disease targets were displayed (Fig. 4A). Then, we incorporated the intersection target genes at the STRING website (https://string-db.org/). The non-intersected targets were set to be hidden, and “highest confidence > 0.900” was set for lowest interaction score; besides, protein-protein interaction (PPI) results were output (Fig. 4B). Using Cytoscape 3.7.2 software, the PPI interaction network of possible anti-ALI therapeutic targets were further obtained. Hub genes were selected with CytoHubba, and those top potential key target genes were screened using the Degree algorithm (Fig. 4C). ALI-LJT-active ingredient-target interaction network was constructed with Cytoscape 3.7.2. Totally 34 nodes (29 targets, 3 active ingredients) and 61 edges were obtained (Fig. 4D).

- Next, R language was applied to perform the analysis of GO annotation and KEGG pathway enrichment on the 156 common targets. The results are shown in Fig. 4E and F. Through GO analysis, 156 potential target genes were suggested to be principally associated with multiple biological processes, including cytokine receptor binding, RNA polymerase II-specific DNA-binding transcription factor binding, and DNA-binding transcription factor binding (Fig. 4E). Using KEGG analysis, it was discovered that above genes were mainly concentrated in IL-17, TNF, and lipid and atherosclerosis pathways (Fig. 4F). In addition, KEGG analysis also suggested that TNF and IL-17 pathways might play their functions by controlling NF-κB pathway (Supplementary Figs. 1 and 2).

### Lut, the major active ingredient of LJT, attenuated LPS-induced ALI and inflammation in vivo

**Fig. 5:**
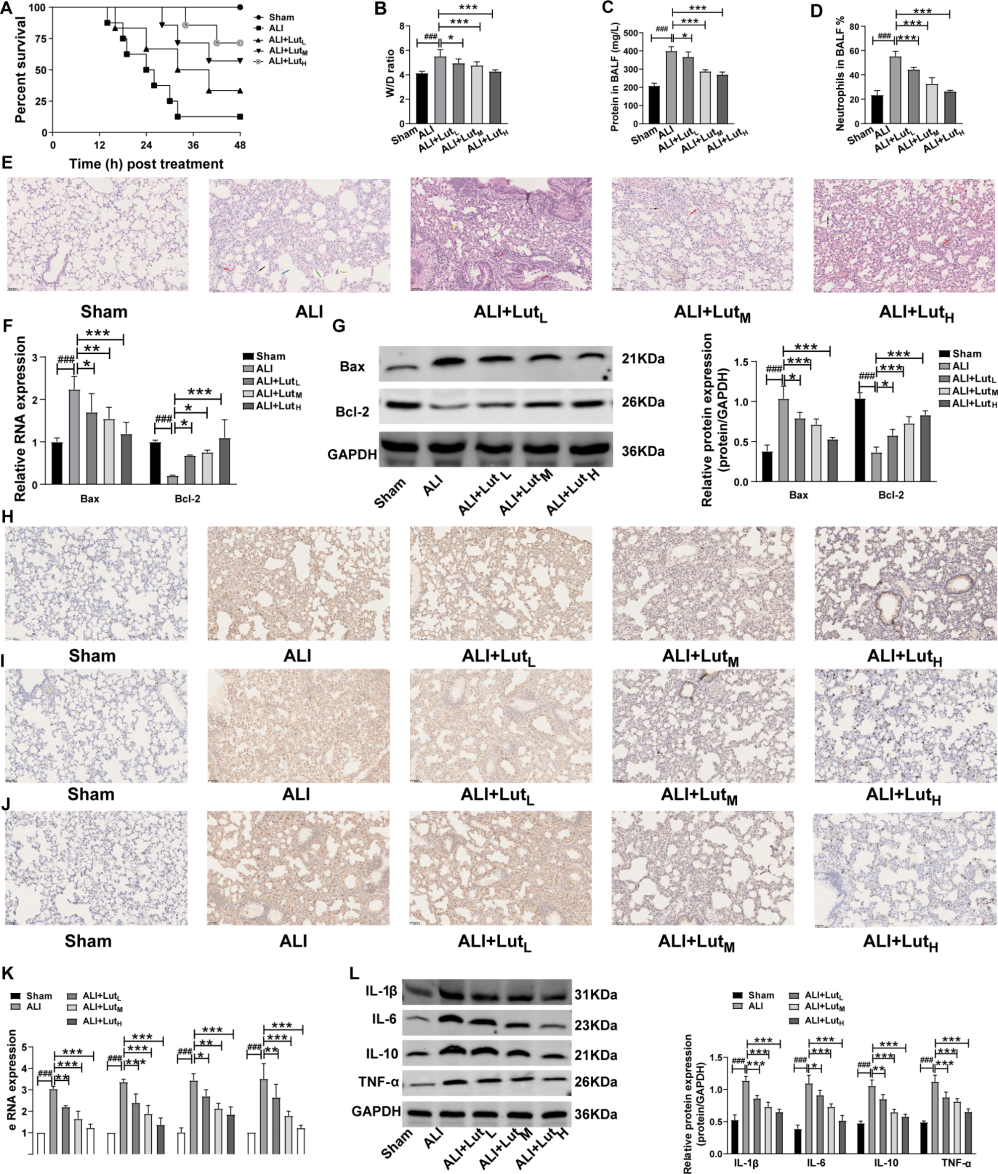
Lut decreased edema, neutrophils, and inflammation in LPS-treated mice. Note:Summarized data representing the effects of different concentrations of Lut on (**A**) the survival overcomes, (**B**) W/D ratio, (**C**) total protein and (**D**) neutrophils in BALF. (**E**) The changes of histological structure of the mouse lung in different groups (20×). (**F**) The mRNA expression levels of Bax and Bcl-2 was tested using qRT-PCR. (**G**) The protein expression levels of Bax and Bcl-2 was tested using western blot assay. The expression levels of of (**H**), IL-1β (**I**) IL-6 and (**J**) TNF-α in mice. (**K**) The mRNA expression levels of IL-1β, IL-6, IL-10 and TNF-α in the tissues of mice were tested by qRT-PCR; (**L**) The protein expression levels of IL-1β, IL-6, IL-10 and TNF-α in the tissues of mice were tested by western blot assays. Bar = 50 μm. ^###^*p* < 0.001 vs. control group; *p < 0.05, **p < 0.01 and ***p < 0.001 vs. ALI model group

To further investigate the influences of Lut on LPS-treated mice, the mice were segmented into Sham, ALI, ALI + Lut_L_ (18 µmol/kg Lut), ALI + Lut_M_ (35 µmol/kg Lut) and ALI + Lut_H_ (70 µmol/kg Lut) groups. At first, the survival outcomes of LPS-induced ALI mice after different treatments were explored by calculating the surviving mouse numbers of the above groups at 48 h after LPS. The results suggested that different concentrations of Lut effectively improved survival rate in LPS-evoked ALI mice compared to ALI mice (Fig. 5A). Meanwhile,the effect of Lut on the lung W/D ratio (an indicator for lung edema in lung tissues) was evaluated. As shown in Fig. 5B, relative to the LPS-stimulated ALI mice, lung W/D ratio was significantly declined in a dose-dependent manner among ALI mice after different concentrations of Lut treatment. Afterwards, the variations of edema, inflammation and neutrophils in BALF were investigated. Compared with Sham mice, LPS-treated mice had increased total protein content and accumulation of neutrophils in the BLAF; the effects were significantly inhibited dose-dependently in mice after Lut or LTE treatment (Fig. 5C and D).

Furthermore,the pathological structures of mice lung tissues in above groups were observed. As expected, the treatment of Lut in different concentrations could notably inhibit the edema, alveolar septal thickening, alveolar and interstitial hemorrhage, as well as infiltration of inflammatory cells in LPS-stimulated ALI mouse lungs (Fig. 5E). Figure 5F and G (Supplementary Table 2) suggested that different concentrations of Lut reversed the function of LPS in the increased Bax expression whereas the reduced Bcl-2 expression in mouse lung tissues. Figure 5H-4J showed that different concentrations of Lut notably inhibited proinflammatory factors levels (IL-1β, IL-6 and TNF-α) in lung tissues from LPS-evoked mice (Supplementary Fig. 3). In addition, Fig. 5K and L (Supplementary Table 2) demonstrated that Lut significantly inhibited the increased RNA and protein levels of the above factors in tissues from LPS-evoked mice in a dose-dependent fashion.

### The effects of Luteolin (Lut) and LTE on the proliferation and apoptosis of LPS-induced BEAS-2B cells

To explore the appropriate concentration of Lut and LTE, the cell viability of LPS-induced BEAS-2B cells after different concentrations of Lut or LTE treatment for 24 h was explored (Supplementary Fig. 4). Finally, 10 µM Lut and 10 µg/mL LTE treatment were chosen for the following study.

**Fig. 6:**
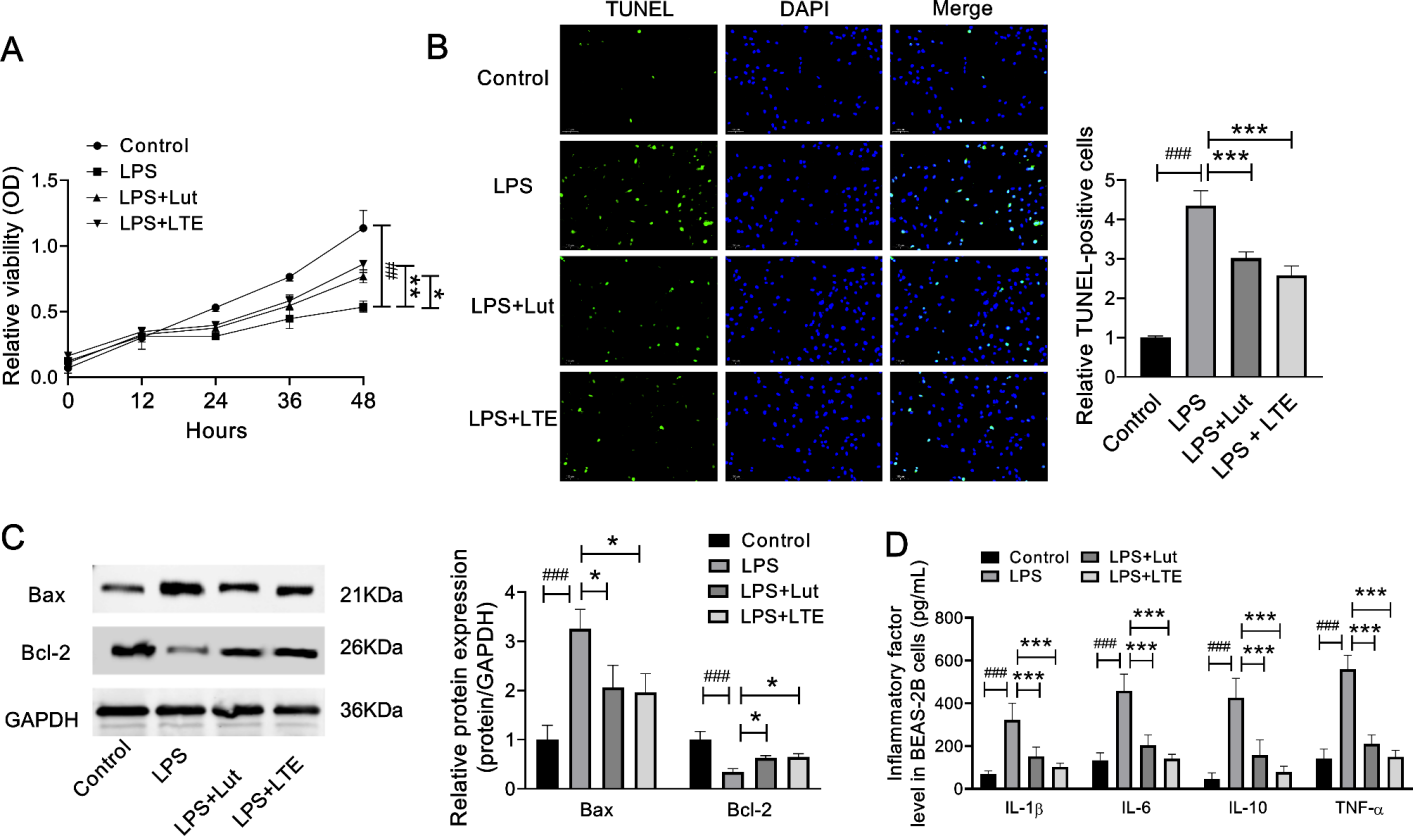
Lut and LTE notably enhanced proliferation and inhibited apoptosis and inflammation in LPS-induced BEAS-2B cells. Note: (**A**) CCK-8 assay was adopted for confirming the impact of Lut and LTE on the proliferation of LPS-induced BEAS-2B cells. (**B**) Cell apoptosis was determined by TUNEL assay in LPS-induced BEAS-2B cells. (**C**) The expression changes of Bax and Bcl-2 were monitored with the use of western blot assay. (**D**) The contents of IL-1β, IL-6, IL-10 and TNF-α in BEAS-2B cells were evaluated using ELISA assay. ^##^*p* < 0.01 and ^###^*p* < 0.001 vs. Control group; *p < 0.05, **p < 0.01 and ***p < 0.001 vs. LPS group

To detect the proliferation of BEAS-2B cells, the CCK-8 assay was performed. Compared to control group, LPS treatment dramatically blocked BEAS-2B cell growth (Fig. 6A). TUNEL assay showed that BEAS-2B cell apoptosis in LPS group was also visibly elevated (Fig. 6B). In addition, the levels of apoptosis-associated markers (Bax, Bcl-2,) in LPS-provoked BEAS-2B cells were investigated by WB and PCR. As displayed in Fig. 6A and C, in relative to control group, LPS induced the enhanced expression of Bax, whereas the reduced expression of Bcl-2. These statistical results demonstrated that the BEAS-2B cell model of ALI was successfully constructed using LPS.

However, such effect was partly reversed by LTE and Lut. Compared with LPS groups, Bax and Cleaved-caspase9 were down-regulated, whereas Bcl-2 was up-regulated in LPS + Lut and LPS + LTE groups.(Figure 6A and C, Supplementary Table 3).

### Lut and LTE alleviated LPS-induced inflammation in vitro models

As previously reported, Lut is a vital anti-inflammatory and anti-oxidant monomer component derived from LJT and makes anti-cancer effects on many types of tumors [21]. Then, we further investigated the inhibitory impacts of Lut and LTE during LPS-mediated inflammation in BEAS-2B cells. In accordance with Fig. 6D, ELISA assay showed that LPS notably elevated IL-1β, IL-6, IL-10 and TNF-α levels in BEAS-2B cells, which were overtly restrained by Lut or LTE, demonstrating that Lut and LTE suppressed LPS-induced inflammation. We disclosed that Lut and LTE could significantly alleviate LPS-mediated BEAS-2B cell injury.

### The inhibition of Lut on the activation of NF-κB pathway was possibly involved in the cell apoptosis in vitro models

The above-mentioned bioinformatics analyses and previous literature indicated that the abnormal activation of NF-κB pathway was the major link and action target of the pathogenesis of ALI. Therefore, the NF-κB signaling pathway-associated protein (MyD88 and IκB-α, P-p65) levels in LPS-provoked BEAS-2B cells were investigated.

**Fig. 7:**
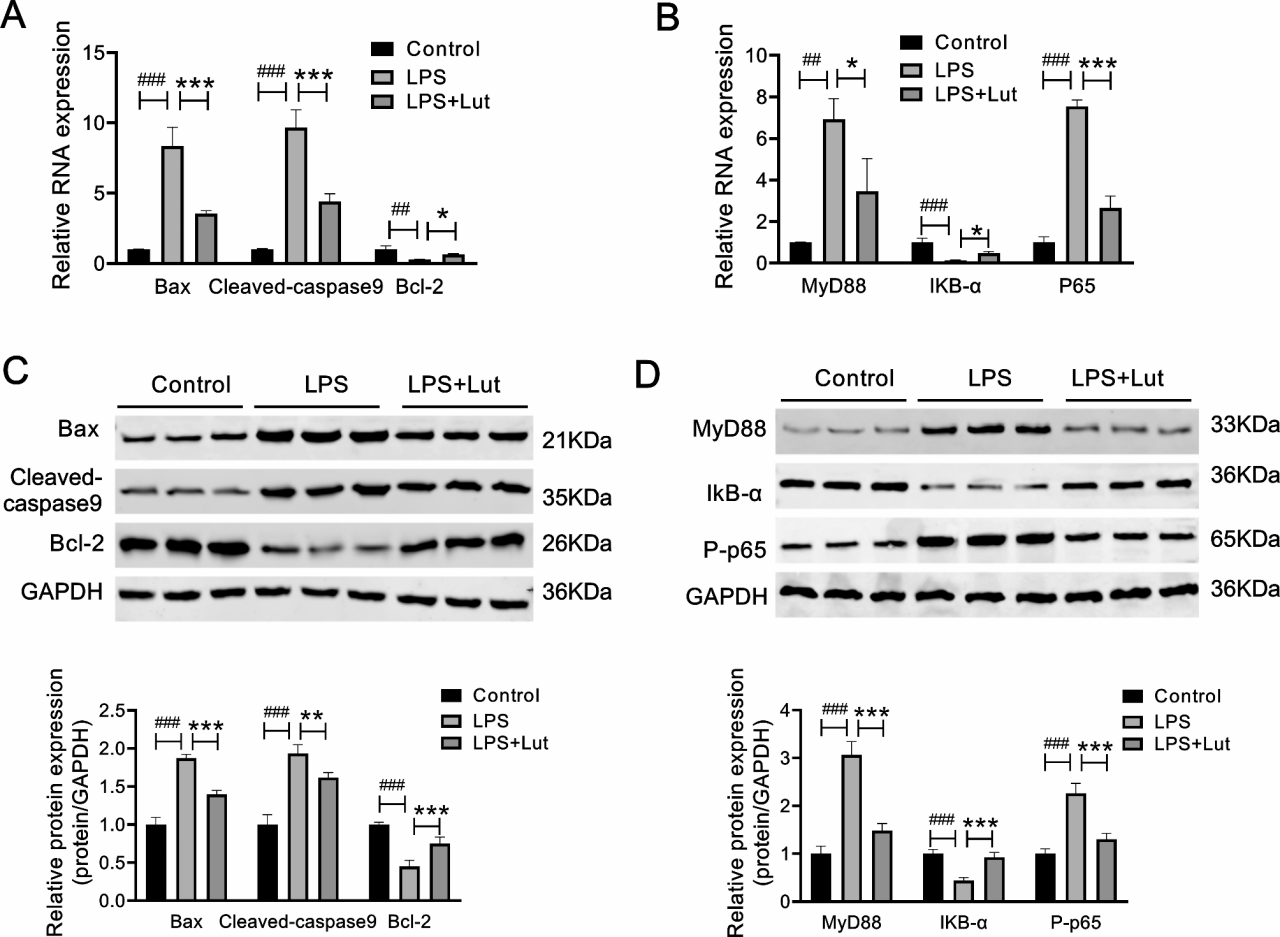
Lut hindered the activation of NF-κB pathway mediated by LPS in BEAS-2B cells. Note:(A and C) BEAS-2B cells were subject to treatment with normal saline, LPS or LPS + Lut. (B and D) The levels of Bax, Bcl-2 and Cleaved-caspase9 were measured by qRT-PCR and western blot assays. The levels of MyD88 and IκB-α and p-p65 were tested with the use of qRT-PCR and western blot assays. ^##^*p* < 0.01, ^###^*p* < 0.001 vs. control group; *p < 0.05, **p < 0.01 and ***p < 0.001 vs. ALI model group

Relative to control group, LPS apparently induced the up-regulation of MyD88 expression and the p65 phosphorylation level, and suppressed IκB-α. While Lut significantly reversed such effect of LPS, down-regulated the LPS-induced MyD88 overexpression and the p65 phosphorylation level, and partially restored the IκB-α expression (Fig. 7B and D, Supplementary Table 4).

As observed from Fig. 7A and C, in relative to normal group, the expression of Bax and Cleaved-caspase9 in LPS-mediated BEAS-2B cells was up-regulated, while Bcl-2 expression was down-regulated. While Lut apparently reversed such effect of LPS, down-regulated the LPS-induced expression of Bax and Cleaved-caspase9 and up-regulated Bcl-2 expression (Supplementary Table 4).

It has been previously indicated that [22, 23], the excessive activation of NF-κB pathway can induce massive cell apoptosis. To further investigate whether BEAS-2B cell apoptosis was related to the activation of NF-κB pathway, we compared the expression of apoptosis-related proteins in the NF-κB pathway-related proteins in BEAS-2B cells. It was seen from Fig. 6A and C that, relative to normal group, Bax and Cleaved-caspase9 expression was up-regulated in LPS-mediated BEAS-2B cells, while Bcl-2 expression was down-regulated. Lut apparently reversed the effect of LPS, down-regulated the LPS-induced expression of Bax and Cleaved-caspase9, and up-regulated Bcl-2 expression. We discovered that, accompanying with the changes in NF-κB pathway-related proteins, the apoptosis-related proteins also show corresponding changes, to be specific, when the NF-κB pathway was suppressed, the apoptosis of BEAS-2B cells decreased, and when the NF-κB pathway was activated, the apoptosis of BEAS-2 cells increased.

The obtained results suggested that Lut might reduce apoptosis triggered by LPS in BEAS-2B cells which maybe associated with restraining MyD88-dependent NF-kB pathway activation (p65 phosphorylation and IkB-α degradation).

## Discussion

LJT, a plant species in traditional Chinese medicine, usually serves to be anti-inflammatory, antiviral, and antipyretic herbal medicine,and It is used to cure the infections of upper respiratory tract. Previous research has reported the protective function of LJT in LPS-evoked ALI in animal models through alleviating inflammatory responses [19]. Nevertheless, the impacts and exact mechanism of LJT on ALI remain unclear. The application of LJT in treating ALI deserves further exploration.

In the current work, the LPS-induced ALI mouse model and the in vitro inflammation model were utilized to explore the mechanisms of LJT against ALI. As observed from Fig. 1, LTE significantly improved the survival rate of ALI rats. Moreover, the results of in vivo study showed that Lut dramatically alleviated the LPS-evoked ALI characteristic pathological changes such as lung pathological injury and lung edema, and exhibited favorable effect against lung injury. Such result was consistent with previous research results [24].

The major pathogenesis of ALI is related to the excessive inflammatory response induced by the cytokine storm [25]. Over-activation of inflammation causes pathological and pathophysiological changes, including pulmonary edema, respiratory membrane thickening, decreased pulmonary surfactant, atelectasis, alveolar wall rupture, alveolar cavity hemorrhage, and imbalanced ventilatory blood flow ratio, leading to respiratory failure, our results consistent with previous results [25]. Our study also revealed that LTE decreased lung W/D rate and total protein content in BALF that were elevated by LPS, which might be correlated with the inhibition of LPS-induced inflammatory storm. As our study also demonstrated that infiltration of inflammation cells and inflammatory reaction were alleviated by LTE treatment in LPS-injured lung tissues. Moreover, our study also discovered that LTE overtly inhibited the indicators related to inflammation, such as inflammatory cell numbers (neutrophils) in lung, which were produced by neutrophils; besides, LTE significantly declined IL-6, IL-1β, TNF-α, and IL-10 levels in BALF of ALI mice and LPS-evoked BEAS-2B cells. Collectively, the above findings showed that LTE could impede LPS-evoked ALI through alleviating the reaction of inflammation in LPS-caused ALI models in vivo and in vitro. Both of our study and previous results indicated that, the advantageous impact of LJT on ALI was largely attributed to its properties in suppressing inflammation [26].

Among the bioactive components of LJT, Lut is a flavonoid compound, which is also an anti-inflammatory polyphenolic medicinal ingredient and is generally found in the plant kingdom [27]. The latest research has testified the prominent suppressive impact of Lut on the inflammation and the progression of disease. For example, Lut reduced the LPS-induced fatal toxicity and expression of pro-inflammatory factors in mice [28]. In addition, Lut blocked prostate cancer cell growth and promoted their apoptosis [17]. Studies have displayed the regulatory effects of Lut on neutrophils [29–31]. Furthermore, Guo et al. suggested that Lut was a key compound of LJT, and could notably lower the secretion of TNF-α of LPS-induced RAW264.7 murine macrophages [32]. Lee et al. indicated that Lut made mitigatory effect on LPS-induced protein leakage and leukocyte infiltration in LPS-induced ALI mice [20]. All the above-mentioned findings suggested that Lut might be beneficial ingredients for alleviating inflammation. Moreover, analysis of the active components of LJT also revealed that Lut might be the major active ingredient of LTE to exert its effect.

Considering the diffuse inflammatory processes of ALI, including the activation or accumulation of neutrophils, and the elevated permeability of blood vessels, this study was aimed at determining whether Lut could alleviate LPS-evoked ALI and explore the latent molecular foundation of LTE. As expected, similar to the function of LTE in LPS-induced mice, Lut treatment also partly mitigated the effect of LPS on the pneumonedema, lung lesions, inflammatory cell infiltration and inflammatory response in LPS-stimulated lung tissues in vivo in a dose-dependent manner. Such result was consistent with previous study [20].

Our studies had demonstrated that Lut could significantly attenuate hemorrhage and pulmonary interstitial edema in LPS-stimulated ALI mouse lung tissues. Therefore, it could be deduced that LTE might alleviate the inflammatory reaction, pulmonary edema and permeability that were stimulated by LPS through active ingredient Lut in mice. Additionally, our results indicated that differences between medium-dose Lut group and high-dose group were not significant, demonstrating that the medium dose of Lut had strong anti-inflammatory effect, which approached the peak. The further increase in dose had limited improvement on the anti-inflammatory effect and might result in drug-related toxic and side effects. Consequently, the medium-dose Lut was selected as the intervention concentration in subsequent cell in vitro experiments.

In vitro, we confirmed that Bax and cleaved-caspase-9 contents increased whereas Bcl-2 contents were downregulated in LPS-evoked BEAS-2B cells in relative to normal groups. However, these effects were all partly restored by Lut stimulation in LPS-evoked BEAS-2B cells. The finding of the in vitro research also demonstrated that different concentrations of Lut visibly facilitated cell viability and restrained cell apoptosis. Relative to LTE group, we discovered that medium-dose Lut exerted the cell protective effect, similar to LTE. This, on one hand, reminded that it was appropriate to select the medium dose of Lut. On the other hand, it indicated that Lut might be the major core active ingredient of LTE to exert its effect, and that the therapeutic effect of LTE might be related to Lut. Then, we preliminarily probed the latent mechanism of Lut in LPS-stimulated ALI.

Active components of LJT and their target proteins were screened from TCMSP. GO and KEGG analyses revealed that the NF-κB signaling pathway might participate in the LTE-mediated protection against LPS-induced ALI. Previous studies have suggested that activating NF-κB signal can promote inflammatory reaction and then accelerate ALI progression [33, 34]. Guo et al. suggested that LJT water extract (FLJWE) and its main ingredient Lut reduced the production of inflammatory and pro-inflammatory factors by suppressing the activation of nuclear factor (NF)-κB in pseudorabies virus (PRV)-infected RAW264.7 cells [35]. Lut could inhibit NF-κB effect on LPS-evoked RAW264.7 cells while mitigating ALI in LPS-injected endotoxin mice [14]. Lut suppressed TNF-α, IL-6 and IL-8 generation through inactivating MAPKs and NF-kB pathways in HMC-1 cells [36]. The underlying mechanism for the protective function of Lut in LPS-stimulated ALI might be related to its impediment on LPS-inducible activation of NF-κB pathway.

Therefore, we explored the levels of apoptosis-associated protein markers (Bax, Bcl-2, cleaved-caspase-9), inflammation-associated markers (MyD88 and IκB-α) and NF-κB signaling pathway-associated protein (P-p65) in LPS-evoked BEAS-2B cells. We also confirmed that Myd88 and p-p65 contents increased whereas IκB-α contents were downregulated in LPS-evoked BEAS-2B cells in relative to normal groups. However, these effects were all partly restored by Lut stimulation in LPS-evoked BEAS-2B cells. Consequently, our results in this study indicated that Lut suppressed the activation of NF-κB signaling pathway, and that its suppression on the NF-κB signaling pathway was partially correlated with the suppression on cell apoptosis. While NF-κB signaling pathway is a key link in the inflammatory response, and the suppression of Lut on inflammation may be related to suppressing this pathway. Thus, it could be speculated that Lut might reduce apoptosis and inflammation reaction induced by LPS in BEAS-2B cells by inactivating MyD88-dependent NF-κB pathway.

Our report revealed that LTE and Lut could both exert anti-inflammatory role in ameliorating LPS-evoked ALI, which might be correlated with the Lut-mediated downregulation of NF-κB signaling pathway. Moreover, this study emphasizing the therapeutical possibility of LTE for ALI, while Lut was its major active ingredient. However, several limitations should be noted in the present study. This research mainly focused on investigating the protective effects of Lut against LPS-induced ALI, whereas there was lack of research on the precise mechanism of action that need to be validated in further research. And the specific mechanisms were still unclear without interventional experiments to validate the pharmacoprotective effects.

### Electronic supplementary material

Below is the link to the electronic supplementary material.

Supplementary Material 1

Supplementary Material 2

Supplementary Material 3

## Data Availability

The datasets used and/or analysed during the current study are available from the corresponding author on reasonable request. All methods were carried out in accordance with the relevant guidelines and regulations.
