# Supplementary material for: Lonicera japonica Thunb extract ameliorates lipopolysaccharide-induced acute lung injury associated with luteolin-mediated suppression of NF-κB signaling pathway
Source: J Inflamm (Lond). 2023 Dec 19;20:44. doi: 10.1186/s12950-023-00372-9 (PMC10729360; doi:10.1186/s12950-023-00372-9)
Supplement: Supplementary file 1 — Supplementary Material 1 [file 12950_2023_372_MOESM1_ESM.doc]

**Supplementary Information**

**Supplementary Figure 1** The relationship between IL-17 signaling pathway and NF-κB signaling pathway.

**

**

**Supplementary Figure 2** The relationship between TNF signaling pathway and NF-κB signaling pathway.

**

**

**Supplementary Figure 3** The viability of LPS-induced BEAS-2B cells after different concentrations of Lut or LTE treatment for 24h was tested by CCK-8 assay. ^##^*p* <0.01 and ^###^*p* <0.001 vs. Control group; **p*< 0.05, ***p* <0.01 and ****p* <0.001 vs. LPS group; ns, no significance.


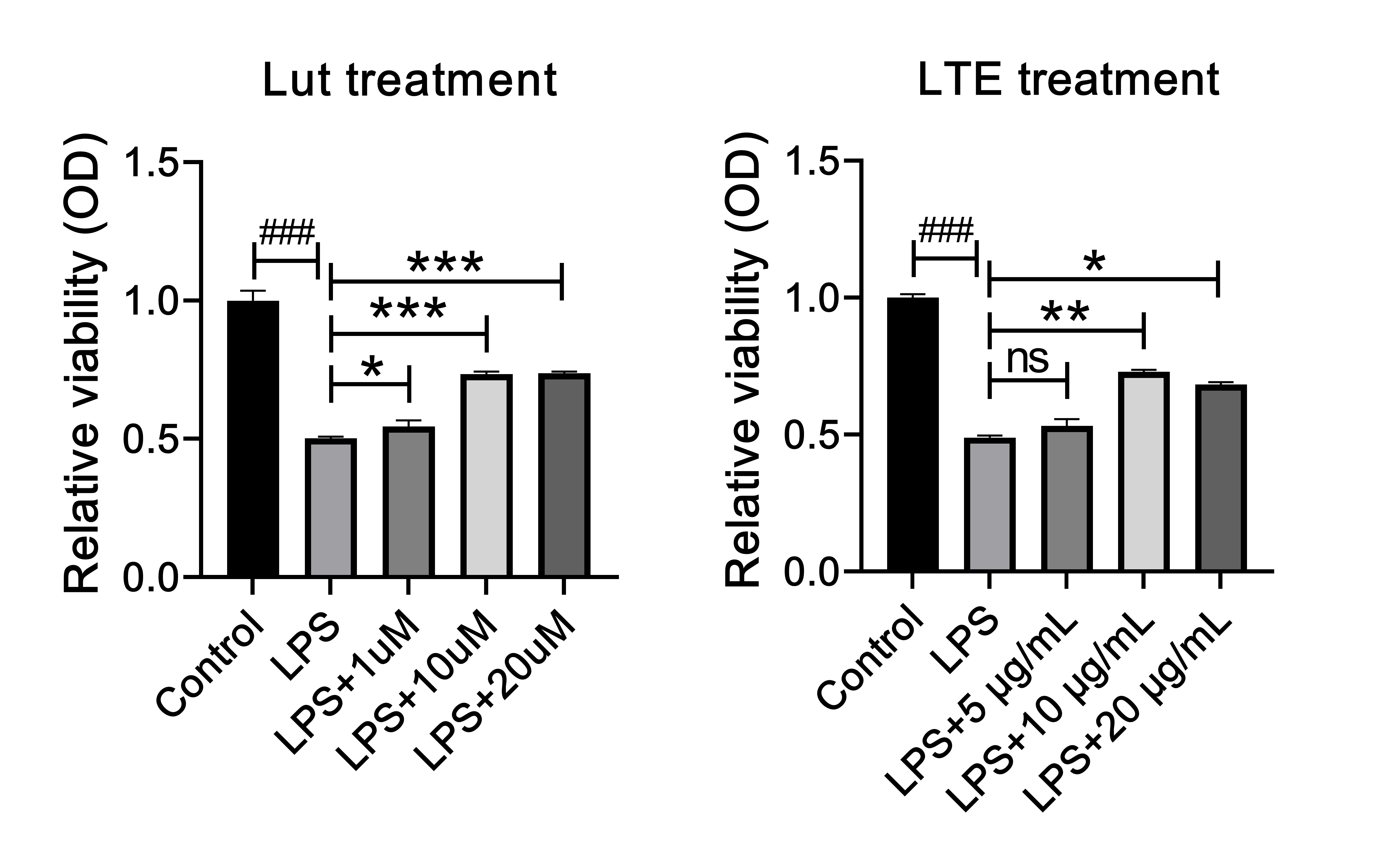


**Supplementary Figure 4** OD values of IL-1β (F), IL-6 (G) and TNF-α (H) in mice detected through IHC assays

**
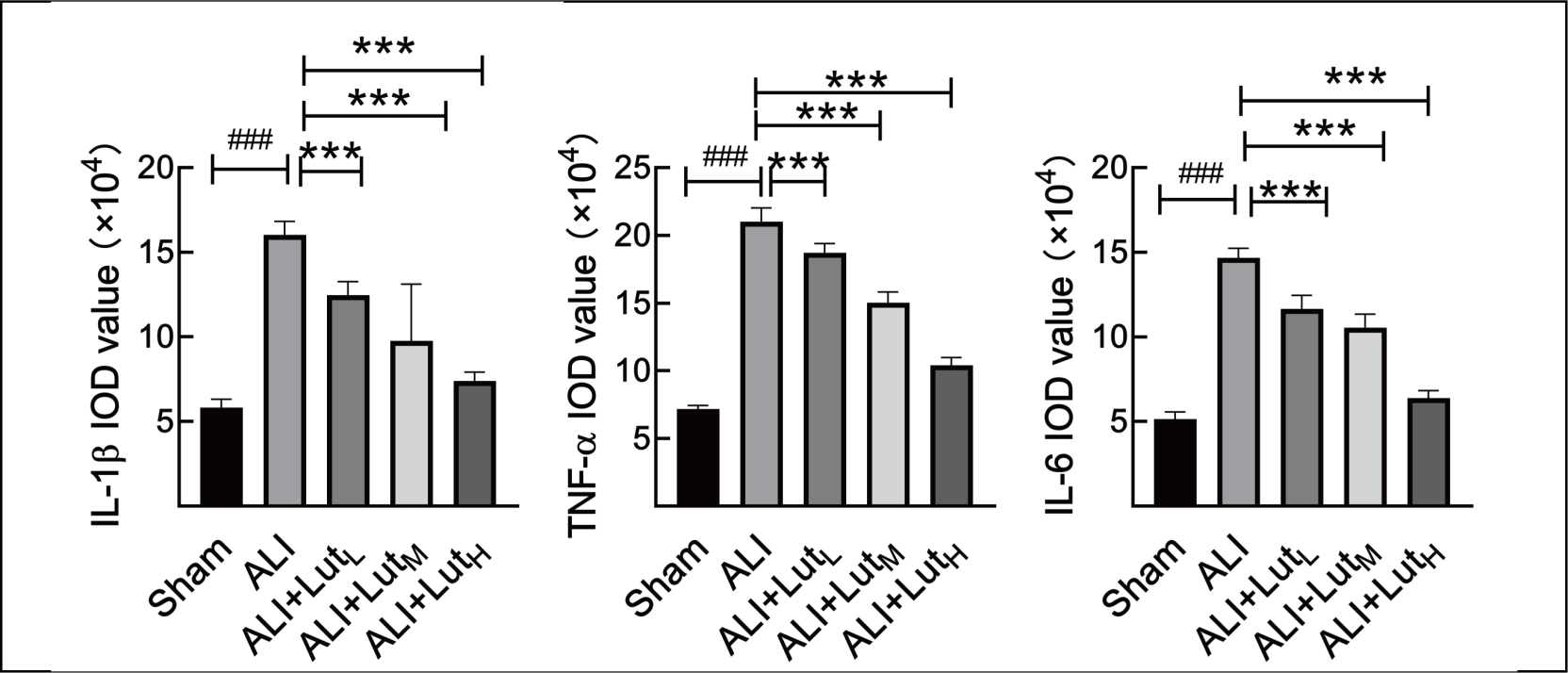
**
