# Supplementary material for: Lonicera japonica Thunb extract ameliorates lipopolysaccharide-induced acute lung injury associated with luteolin-mediated suppression of NF-κB signaling pathway
Source: J Inflamm (Lond). 2023 Dec 19;20:44. doi: 10.1186/s12950-023-00372-9 (PMC10729360; doi:10.1186/s12950-023-00372-9)
Supplement: Supplementary file 2 — Supplementary Material 2 [file 12950_2023_372_MOESM2_ESM.doc]

**Supplementary Table 1.（Protein/GAPDH）Relative protein expression quantities of inflammatory factors after LTE treatment (Protein/GAPDH)**

|  | Sham | ALI | ALI+LTE |
| --- | --- | --- | --- |
| IL-1β | 1.00±0.40 | 3.38±0.51 | 2.21±0.33 |
| IL-6 | 1.00±0.11 | 3.38±0.35 | 2.51±0.33 |
| IL-10 | 1.00±0.29 | 2.14±0.18 | 1.57±0.17 |
| TNF-α | 1.00±0.57 | 2.99±0.53 | 1.76±0.26 |

**Supplementary Table 2.（Protein/GAPDH）Relative protein expression quantities of inflammatory factors after treatment with different concentrations of Lut (Protein/GAPDH)**

|  | Sham | ALI | ALI+LutL | ALI+LutM | ALI+LutH |
| --- | --- | --- | --- | --- | --- |
| IL-1β | 0.53±0.08 | 1.14±0.07 | 0.86±0.05 | 0.73±0.07 | 0.65±0.04 |
| IL-6 | 0.39±0.06 | 1.10±0.13 | 0.91±0.08 | 0.73±0.05 | 0.52±0.08 |
| IL-10 | 0.48±0.03 | 1.06±0.09 | 0.85±0.07 | 0.65±0.05 | 0.58±0.04 |
| TNF-α | 0.49±0.02 | 1.12±0.10 | 0.88±0.08 | 0.81±0.05 | 0.65±0.05 |
| Bax | 0.38±0.08 | 1.04±0.16 | 0.79±0.07 | 0.71±0.07 | 0.53±0.02 |
| Bcl-2 | 1.04±0.07 | 0.37±0.07 | 0.58±0.08 | 0.73±0.08 | 0.83±0.05 |

**Supplementary Table 3. Relative protein expression quantities of Bax and Bcl-2 after LTE/Lut treatment (Protein/GAPDH)**

|  | Control | LPS | LPS+Lut |
| --- | --- | --- | --- |
| Bax | 1.00±0.10 | 1.87±0.05 | 1.40±0.05 |
| Cleaved-caspase9 | 1.00±0.13 | 1.93±0.12 | 1.62±0.06 |
| Bcl-2 | 1.00±0.03 | 0.45±0.08 | 0.75±0.09 |
| MyD88 | 1.00±0.16 | 3.07±0.28 | 1.48±0.15 |
| IKB-α | 1.00±0.09 | 0.44±0.06 | 0.92±0.10 |
| nuclear-p-p65 | 1.00±0.10 | 2.26±0.21 | 1.31±0.12 |

**Supplementary Table 4.（Protein/GAPDH）Relative protein expression quantities of NF-κB proteins and apoptosis-related proteins after Lut treatment (Protein/GAPDH)**

|  | Control | LPS | LPS+Lut | LPS+LTE |
| --- | --- | --- | --- | --- |
| Bax | 1.00±0.29 | 3.26±0.39 | 2.06±0.45 | 1.96±0.38 |
| Bcl-2 | 1.00±0.16 | 0.34±0.07 | 0.63±0.05 | 0.65±0.06 |
