# Supplementary material for: Lonicera japonica Thunb extract ameliorates lipopolysaccharide-induced acute lung injury associated with luteolin-mediated suppression of NF-κB signaling pathway
Source: J Inflamm (Lond). 2023 Dec 19;20:44. doi: 10.1186/s12950-023-00372-9 (PMC10729360; doi:10.1186/s12950-023-00372-9)

**Supplementary Material**

**Original gels and blot images**

Figure.3 Protein expression levels of IL-1β in the tissues of mice evaluated by western blot assays


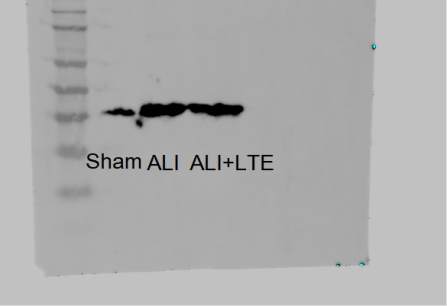


Figure.3 Protein expression levels of IL-6 in the tissues of mice evaluated by western blot assays


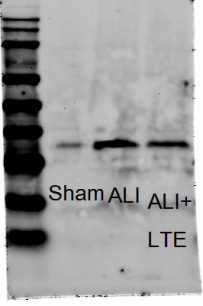


Figure.3 Protein expression levels of IL-10 in the tissues of mice evaluated by western blot assays


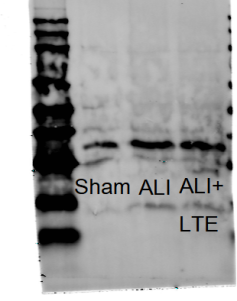


Figure.3 Protein expression levels of TNF-α in the tissues of mice evaluated by western blot assays


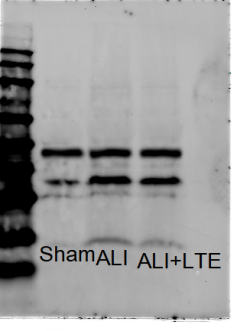


Figure.5 (L) The protein expression levels of IL-1β in the tissues of mic tested by western blot assays


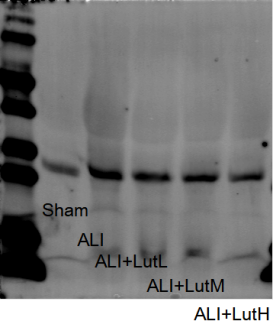


Figure.5 (L) The protein expression levels of IL-6 in the tissues of mic tested by western blot assays


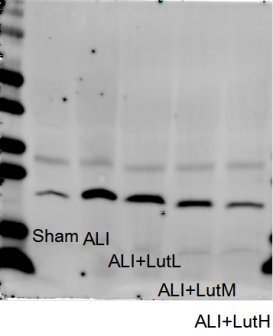


Figure.5 (L) The protein expression levels of IL-10 in the tissues of mic tested by western blot assays


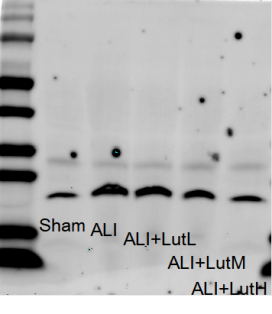


Figure.5 (L) The protein expression levels of TNF-α in the tissues of mic tested by western blot assays


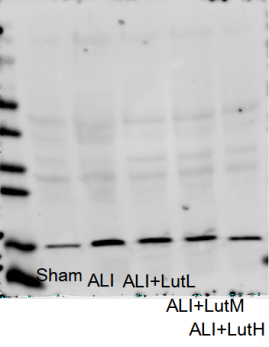


Figure.5 (G) The protein expression levels of GAPDH in the tissues of mic tested by western blot assays


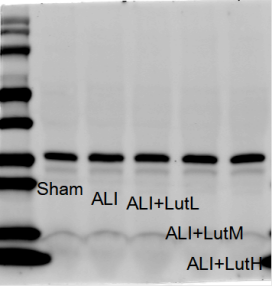


Figure.5 (G) The expression levels of Bax tested by western blot assays


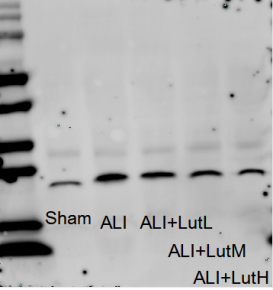


Figure.5 (G) The expression levels of Bcl-2 tested by western blot assays


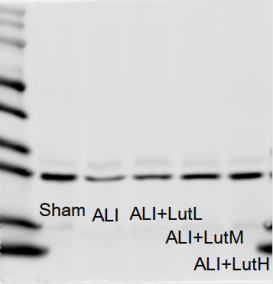


Figure.5 (L) The expression levels of GAPDH tested by western blot assays


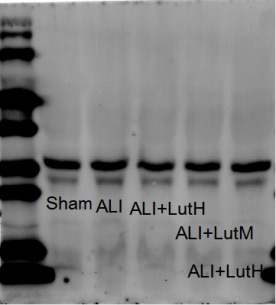


Figure.6 The expression changes of Bax were monitored with the use of western blot assay


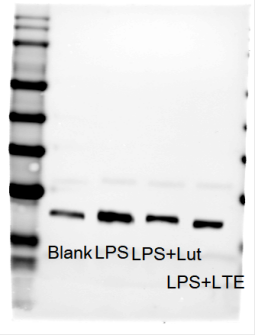


Figure.6 The expression changes of Bcl-2 were monitored with the use of western blot assay


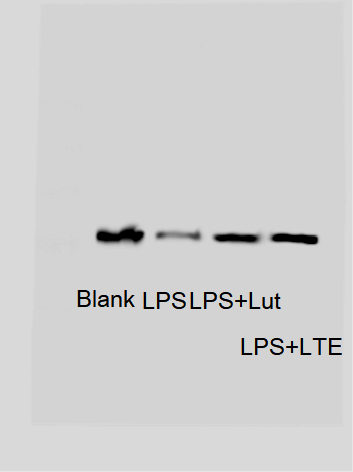


Figure.6 The expression changes of GAPDH were monitored with the use of western blot assay


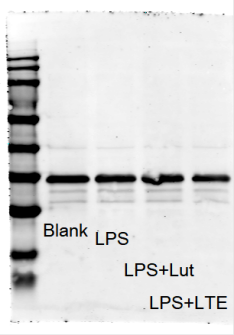


Figure.7 (C) The levels of Bax measured by western blot assays


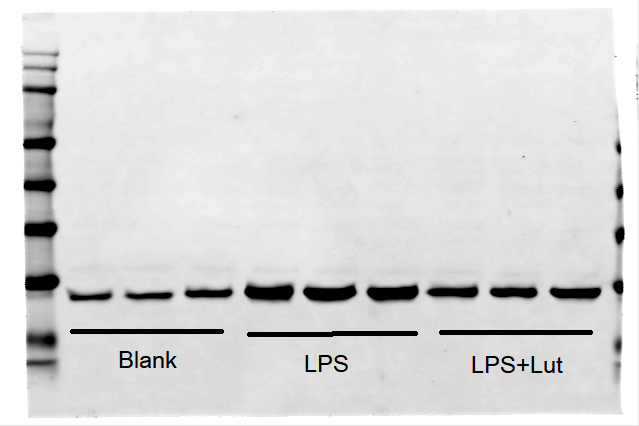


Figure.7 (C) The levels of Bcl-2 measured by western blot assays


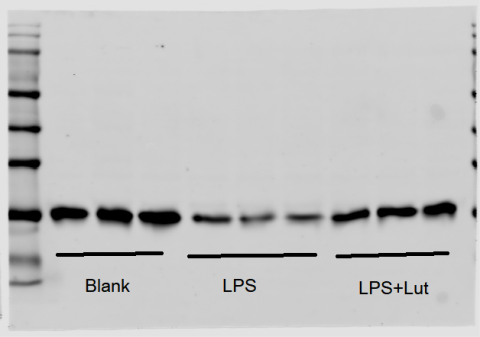


Figure.7 (C) The levels of Cleaved-caspase9 measured by western blot assays


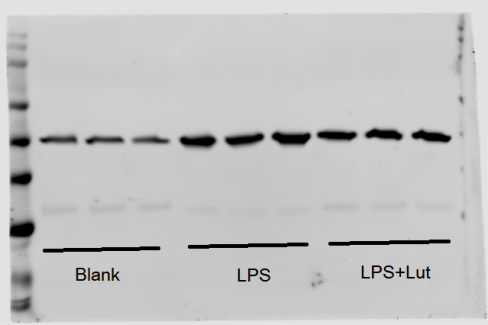


Figure.7 (C) The levels of GAPDH measured by western blot assays


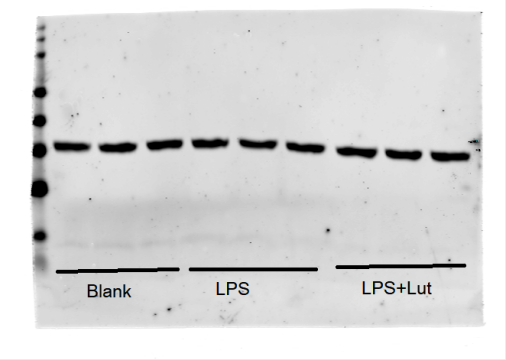


Figure.7 (D) The levels of MyD88 measured by western blot assays


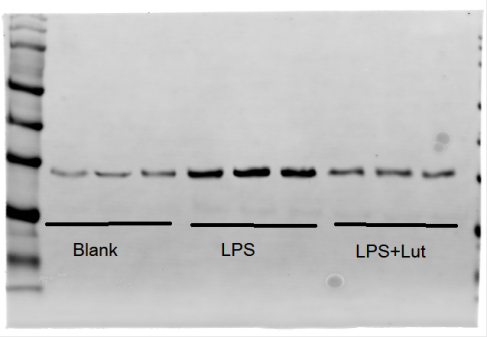


Figure.7 (D) The levels of IκB-α measured by western blot assays


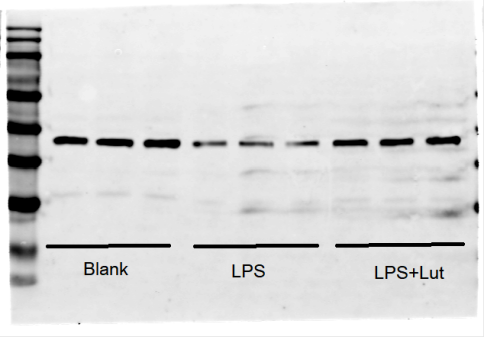


Figure.7 (D) The levels of nuclear p-p65 measured by western blot assays


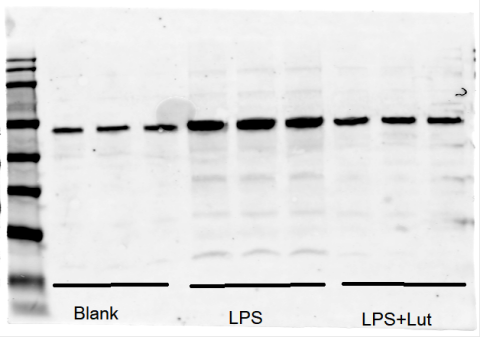


Figure.7 (D) The levels of GAPDH measured by western blot assays


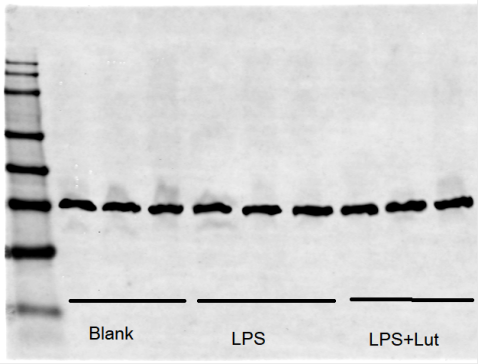

Supplement: Supplementary file 3 — Supplementary Material 3 [file 12950_2023_372_MOESM3_ESM.docx]
